# Essential Oil Variability of Superior Myrtle (*Myrtus communis* L.) Accessions Grown under the Same Conditions

**DOI:** 10.3390/plants11223156

**Published:** 2022-11-18

**Authors:** Donya Shahbazian, Akbar Karami, Fatemeh Raouf Fard, Saeid Eshghi, Filippo Maggi

**Affiliations:** 1 Department of Horticultural Science, School of Agriculture, Shiraz University, Shiraz 71441, Iran; 2 Chemistry Interdisciplinary Project (ChIP), School of Pharmacy, University of Camerino, 62032 Camerino, Italy

**Keywords:** essential oil (EO), myrtle, chemotype, α-pinene, 1,8-cineole, linalyl acetate, accessions

## Abstract

Myrtle (*Myrtus communis* L., Myrtaceae) has numerous applications in pharmacology, food technology, and cosmetic industry. The current research aimed at measuring variations in the leaf essential oil (EO) compositions of 14 superior myrtle accessions originating in natural habitats of south Iran. The plants were grown under greenhouse conditions. Fresh leaf samples were harvested in June 2021. Based on dry matter, the extractable amount of EO in the accessions ranged from 0.42% (BN2) to 2.6% (BN5). According to GC/MS analysis, the major compounds in the EO were α-pinene (2.35–53.09%), linalyl acetate (0–45.3%), caryophyllene oxide (0.97–21.8%), germacrene D (0–19.19%), α-humulene (0–18.97%), 1,8-cineole (0–18.0%), limonene (0–17.4%), and *p*-cymene (0–13.2%). These myrtle accessions were classified into four groups, including I: caryophyllene oxide/germacrene D/α-humulene/methyl eugenol chemotype; II: α-pinene/*p*-cymene/α-humulene and (*E*)-β-caryophyllene; III: α-pinene/1,8-cineole, and linalool; IV: linalyl acetate/γ-terpinene/1,8, cineole/limonene. These classifications were established by considering the main EO components using hierarchical cluster analysis (HCA) and principal component analysis (PCA). In summary, this study provided new insights into available opportunities of selecting suitable genotypes for commercial cultivation purposes and planning breeding programs in the future.

## 1. Introduction

Myrtle (*Myrtus communis* L.) is a valuable species within fragrant medicinal plants of the Myrtaceae family. It is an evergreen shrub that grows wild in many regions of the world and is widely cultivated. Commercial plantations of this species can be found in southern Europe, southern Africa, and the Middle East [1,2]. The fruits of myrtle are usually found in two colors: white and deep blue [2]. The white variant is far more uncommon and is distinguished by its unique leaves. The species is biologically characterized by poor pollen, weak seed distribution, and low environmental adaption, which may account for its rarity [3]. As a prized shrub, it has been used for centuries because of its antibacterial, hypoglycemic, and antiseptic properties [3,4,5,6]. Myrtle essential oil (EO) is now used in pharmacology, food technology, and the cosmetic industry [7]. As a matter of its emollient qualities, myrtle is used in conventional medicinal applications [8]. Noteworthy, myrtle EO functions as a nematicide, insecticide, antibacterial, and fungicide [4]. Numerous cases of research have led to descriptions of the chemical makeup of myrtle in several countries, including Tunisia [3,4,5,9,10,11], Algeria [12,13], Albania [14], Italy [8], Iran [2,15,16,17,18], Greece [19], Egypt [20], Yemen [21] and Turkey [22]. The species is recognized for its variable chemical compositions in different countries.

Developing new products for the pharmaceutical and nutraceutical industries may require crucial assessment of variations in EO chemical profiles among medicinal plants. The EO content and composition are significantly influenced by genetic factors and environmental conditions such as soil characteristics, temperature, precipitation, and altitude [1,2,3,4,8,10,17]. Therefore, valuable findings may result from comparison between various plants of the same species that originate in different regions. Their EO contents and compositions can be analyzed for diversity and, thus, provide researchers with new insights into the specifics of desirable EO components. So, in the current study, we measure variations in the leaf essential oil (EO) compositions of 14 superior myrtle accessions originating in natural habitats of south Iran and grown under greenhouse conditions.

## 2. Results and Discussion

### 2.1. Essential Oil Content of Myrtle Accessions under Greenhouse Conditions

The 14 myrtle accessions showed significant variations in EO content (*p* < 0.01). Based on dry weight, the EO yields of the 14 myrtle accessions in greenhouse conditions ranged from 0.42% (BN2) to 2.6% (BN5) (Table 1). Previous research indicated that myrtle EOs in the Fars province ranged from 0.53% in Polemord to 1.75% in Bajgah populations [2]. This demonstrated that myrtle populations in the Fars province are identifiable by their extraordinary valuable EOs. The EO yields of Iranian myrtle leaves reportedly vary between 0.6 and 1.4 mL/100 g, which is within the range observed in the current study [17]. Mimica-Duki’c et al. [23] reported EO yields of 0.72–0.81% in myrtle plants harvested from Montenegro. Furthermore, EO yields of Tunisian myrtles ranged from 0.44 to 0.6% [3,11].

### 2.2. EO Compositions of Myrtle Accessions under Greenhouse Conditions

EO components were evaluated in 14 myrtle accessions. Apart from trace elements, all other compounds were identified (Table 1). In the Fars province, the myrtle accessions showed substantial variations in chemical profile. A total of 23 chemical compounds was identified in the myrtle accessions analyzed. The majority of the compounds accounted for α-pinene (2.35–53.09%), linalyl acetate (0–45.3%), caryophyllene oxide (0.97–21.8%), germacrene D (0–19.19%), α-humulene (0–18.97%), 1,8-cineole (0–18.0%), limonene (0–17.4%), and *p*-cymene (0–13.2%). In terms of percentages, large differences existed among the identified major compounds in the myrtle EOs of the different accessions. The accessions of KH3, AT1, AT2, AT5, BN5, KA1, KA3, and KA4 showed maximum amounts of α-pinene (16.3–53.09%). The lowest amounts of α-pinene were observed in the EOs of accessions AT3 (2.35%), AT4 (2.63%), and KH1 (3.21%) (Table 1). Maximum and minimum values of linalyl acetate were observed in accessions AT3 (45.3%), AT2 and AT4 (undetectable amounts), respectively. Maximum and minimum values of caryophyllene oxide belonged to AT4 (21.8%) and BN5 (0.97%), respectively. The highest percentages of 1,8-cineole were observed in accessions KH1 (18.1%), KA4 (17.64%), and KA3 (17.4%). The lowest percentage of this compound was observed in accessions AT4 and BN1. According to Table 1, except for AT4 and BN1, all accessions revealed substantial percentages of 1,8-cineole. Large variations were observed in limonene content among the different accessions (from 1.6% in AT5 to more than 17.4% in BN2).

There are several reports of EOs obtained from myrtle accessions from different countries. Nonetheless, there have been few considerations in Iran to study the available diversity in different topographical districts. Rahimmalek et al. [17] described changes in the chemical composition of myrtle populations in Iran, although the measurements did not cover the traits measured in the current research. These populations primarily consisted of 1,8-cineole, limonene, linalool, α-terpineol, and linalyl acetate. Additionally, inhabitants in the Fars region were found to have a significant concentration of α-pinene (34.58–47.83%).

Regarding 1,8-cineole in the EOs, the lowest and highest percentages were found in Lo4 (9.56%) and Fa4 (28.94%). Myrtle populations from southwestern Iran were previously studied according to Yadegarinia et al. [18]. The main substances identified in this research were linalool (10.4%), 1,8-cineole (17.9%), α-pinene (29.1%), and limonene (21.5%). In addition, Ghasemi et al. [13] revealed that myrtle populations in Manjil, Iran, had significant percentages of limonene (15%), 1,8-cineole (23%), and α-pinene (31%).

As already expected, previous reports indicated diverse variations in the amounts of linalool, α-pinene, 1,8-cineole, limonene, and myrtenyl acetate in the EOs of myrtle accessions from different parts of the world. Thus, the current data in the available literature is specifically tailored to each region. According to Yangui et al. [4], linalool, myrtenyl acetate, α-pinene, and 1,8-cineole were identified as principal components in the EOs of Tunisian myrtle. It was reported that myrtle populations of Boussalem and Oueslatia had the highest levels of limonene and myrtenyl acetate, while samples of the Zaghouan region were distinguished for their high linalool content. In line with the current study, Wannes et al. [9] reported that ZF and KFW populations of *M. communis* var. *italica* had the highest percentagesì of α-pinene (58.5%) in the leaf, whereas Messaoud et al. [3] measured 12.24–30.7% of α-pinene in myrtle genotypes of different Tunisian populations. According to Berka-Zougali et al. [12], the most significant components of Algerian myrtle were 1,8-cineole (25.46%) and α-pinene (44.62%). Flamini et al. [8] reported a high amount of 1,8-cineole (52.7%) in Italian myrtles. Alike, in Egypt, the major EO component in the leaves of myrtle was 1,8-cineole (27.2%) [20]. Messaoud et al. [3] reported that camphene, 1,8-cineole, and α-pinene were major EO components in Tunisian myrtles. In the current research, the major EO component percentages varied, with notable amounts of α-pinene (2.35–53.09%), linalyl acetate (0–45.3%), caryophyllene oxide (0.97–21.8%), and germacrene D. In earlier research [8,24], the α-pinene content was reportedly significant in myrtle berries. According to Messaoud and Boussaid [5], two myrtle morphs were evaluated phytochemically in their berries for EO contents. In the said research, it was reported that dark blue berries were more likely to have higher amounts of α-terpineol, linalool, α-pinene, methyl eugenol, and geraniol, whereas white fruits primarily contained myrtenyl acetate.

### 2.3. Cluster Analysis of the Myrtle Chemotypes Based on the Main Compounds

**Figure 1:**
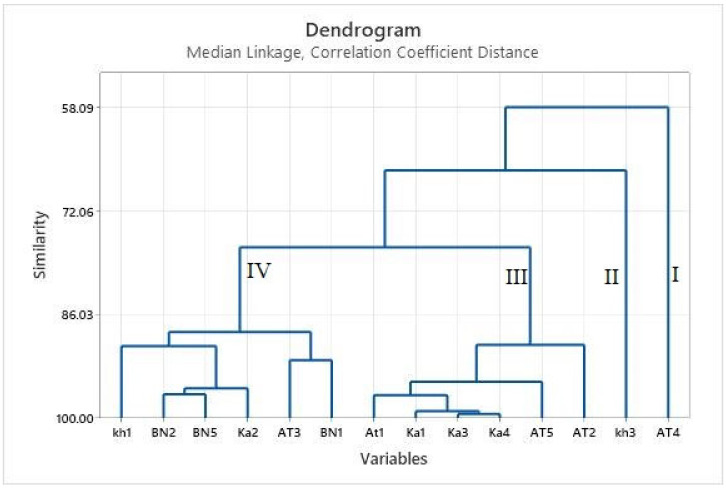
Cluster analysis of 14 superior myrtle accessions from southern Iran under greenhouse conditions. This analysis based on their major essential oil (EO) composition using Median Linkage method. The myrtle accessions were classified into 4 groups based on the main EO components, including I: caryophyllene oxide/germacrene D/α-humulene/methyl eugenol chemotype; II: α-pinene/*p*-cymene/α-humulene and (*E*)-β-caryophyllene; III: α-pinene/1,8-cineole, and linalool; IV: linalyl acetate/γ-terpinene/1,8-cineole/limonene.

Cluster analysis was carried out on the populations to distinguish between the potential groups. While 23 compounds were discovered, a cluster analysis using the median linkage method revealed four distinct groups in the dendrogram (Figure 1).

The first group had AT4 accession only and was unique for its large percentages of caryophyllene oxide, germacrene D, α-humulene, and methyl eugenol. Additionally, it was poor in limonene and 1,8-cineole (caryophyllene oxide/germacrene D/α-humulene/methyl eugenol chemotype). This accession was grouped separately for having large percentages of caryophyllene oxide. The only member of the second group was KH3, which showed high levels of *p*-cymene, α-humulene, and (*E*)-β-caryophyllene (chemotype α-pinene/*p*-cymene/α-humulene and (*E*)-β-caryophyllene). The third group, which included chemotypes AT2, AT5, KA4, KA3, KA1 and AT1, was distinguished by significant percentages of 1,8-cineole, α-pinene, and linalool. According to the dendrogram, the fourth group consisted of the accessions BN1, AT3, KA2, BN5, BN2, and KH1, which were distinguished by high concentrations of linalyl acetate/γ-terpinene/1,8-cineole/limonene. Since this accession had a high percentage of linalyl acetate, it was separated from the other accessions. Iranian populations of myrtle were identified by various chemicals in earlier studies [2]. ZF and KFW populations had the highest percentage of α-pinene. The populations of KA, AT1, and AT2 contained the largest percentage of 1,8-cineole. More than 26% of the highest limonene percentage was found in the PN population. In the TN population, a rather high percentage of linalool (21% and more) was noted. Meanwhile, the myrtenyl acetate percentage was greatest in the ESH population. [2]. Numerous cases of research [2,3,4,5,6,7,8,9,10,11,12,13,14,15,16] considered the chemical makeup of myrtle EO. Since EOs are usually complex combinations, a range of factors can shape their chemical compositions. Ecological conditions, genetic factors, and plant ontology are among the main factors that determine the chemical composition of myrtle EO [4]. In previous research by Bradesi et al. [25], myrtle genotypes were categorized into two primary chemotypes based on their geographic origin and myrtenyl acetate concentration. The Oueslatia was the only accession that could be associated with the myrtenyl acetate chemotype in a different study on Tunisian myrtle, whereas the myrtle genotypes collected from Nefza, Sejnene, Zaghouan, and Boussalem were linked to the α-pinene/1,8-cineole chemotype [4]. In a similar study, an analysis of myrtle EO revealed at least 12 unique chemotypes, with reference to a hierarchical cluster analysis of chemical compositions of 98 myrtle samples in the available literature and one sample from Yemen [21]. Only the myrtle EO of ESH corresponded with the myrtenyl acetate/linalool/limonene chemotype in a previous study on Iranian myrtle, whereas the myrtle EO of the PN population resembled the α-pinene/limonene/1,8-cineole chemotype. Other populations in the Fars province of Iran belonged to the 1,8-cineole/α-pinene and 1,8-cineole/α-pinene/linalool chemotypes [2]. Rahimmalek et al. [17] described the α-pinene/1,8-cineole chemotypes of Iranian myrtle. In particular, four chemotypes of Iranian myrtle were mentioned in another study [16], including chemotypes of α-pinene/1,8-cineole/linalool, α-pinene/linalool, α-pinene/1,8-cineole, and α-pinene/1,8-cineole/limonene. In the current research, Iranian myrtles were grown under greenhouse conditions and showed significant percentages of linalyl acetate. The other ecological site of experiment was home to different chemotypes in nearby locations such as Shiraz, Firozabad, and Noor Abad, thereby confirming previous findings by Messaoud et al. [3]. The current research demonstrated that the role of genetic factors was more prominent than the role of ecological conditions in causing the differences among the EOs. Therefore, genotypes can be selected for cultivation based on their chemical compositions.

### 2.4. Principal Component Analysis (PCA)

**Figure 2:**
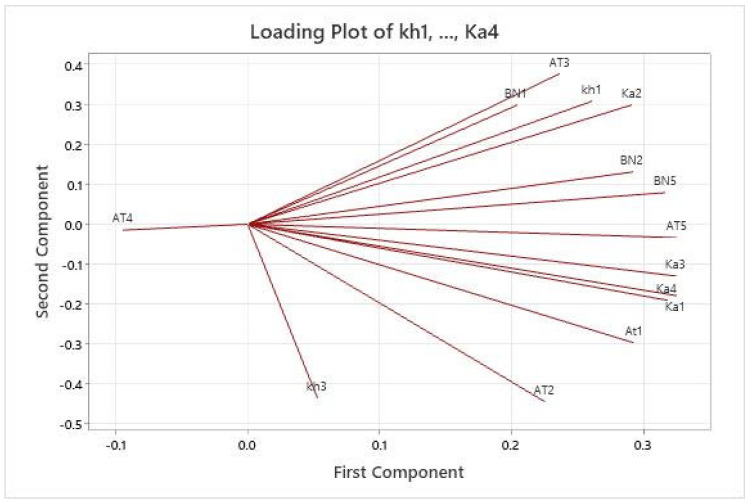
Biplot of the first two principle components (PCs) for the studied accessions of myrtle under greenhouse conditions.

The main EO compounds and accessions were selected for principal component analysis using a correlation matrix (PCA). Figure 2 displays the eigenvalues and total variance for each factor. According to the PCA, the cluster analysis was confirmed and the results of HCA were validated.

## 3. Materials and Methods

### 3.1. Plant Materials and Site Description

In June 2021, leaf samples were taken from the suckers of 14 superior myrtle accessions that grew in greenhouse conditions. The accessions naturally originated in Fars province, Iran (Table 2). The samples were harvested according to a method described by Shahbazian et al. [2]. Using a Global Positioning System (GPS) (Vista Garmin, Olathe, KS, USA), the locations of the plants were recorded. After labeling each plant sample, they were transferred to greenhouse conditions for acclimation and further analysis (day/night 16/8 h, light intensity 150 μmol m^−2^ s^−1^, 28 ± 2 °C/22 ± 2 °C day/night, and relative humidity 55–75%). Table 2 reports several characteristics of the samples at the site of collection.

### 3.2. EO Isolation and Phytochemical Analysis

Six days before the extraction of EO, fresh leaves were collected and placed in the open air, under a shade, to be naturally air-dried. For hydro-distillation, dried leaves (100 g) were used, following their immersion in distilled water (1000 mL). A Clevenger-type apparatus functioned for the extraction of EOs for 3 h. This was followed by collecting the EOs in a container and determining the EO contents based on dry matter. The variables were measured in three replicates. The EOs were dried on anhydrous sodium sulfate and subsequently stored at 4 °C. When appropriate, they were dissolved in *n*-hexane (Merck) for Gas Chromatography/Mass Spectrometry (GC/MS) analysis.

GC analysis was carried out on an Agilent 7890-A GC. The descriptions of the device and the mode of function were similar to those reported in an earlier study by Shahbazian et al. The specifics that were identical to the earlier research included descriptions of film thickness, Flame Ionization Detector (FID), temperature of the injector and detector, carrier gas and its flow rate, the dilution of EO, and injections in split mode. The split ratio and the program for the increase in oven temperature were also borrowed from a relevant procedure described by Shahbazin et al. [2]

The GC/MS analysis was carried out using the same Agilent gas chromatograph in association with a mass spectrometer detector (Model 5975 C) and an HP-5 MS-fused capillary column of silica (film thickness 0.25 μm). The carrier gas was helium. Temperatures of 230 °C and 280 °C were considered for the ion source and the contact, respectively. Column thermal program began at 60 °C, increased to 210 °C with the rate of 3 °C/min and then reached 240 °C with 20 °C/min rate. The program was maintained at the latter temperature for 8.5 min. The injector temperature used in this study was 280 °C. A 70 eV ionization voltage was used. Values between 45 and 550 amu were selected as the mass range. An identical oven temperature program that was used for GC FID was applied herein.

While applying an *n*-alkane mixture (C_8_–C_25_) under chromatographic conditions as mentioned previously, the Retention Indices (RIs) of EO components were programmed for the increase in temperature. Computations and comparisons were made according to relevant protocols in the available literature [26]. Comparisons of mass spectra mimicked published values in libraries of mass spectral indications, including those from the National Institute of Standards and Technology’s NIST 08 and the Wiley/ChemStation data system. In the absence of correction variables, relative area percentages were acquired using FID and then used for quantification.

### 3.3. Statistical Analyses

Comparison among the EO contents carrying out a 3-replicate in the completely randomized design was used and mean comparisons were made using Fisher’s Least Significant Difference (LSD). The mean values were considered to have statistical significance at *p* < 0.05. The cluster analysis was carried out according to the median-linkage method in SPSS software, and the PCA analyses were studied by Minitab software (V.21.1).

## 4. Conclusions

According to the current study, Iranian accessions of myrtle exhibited a great diversity in terms of chemical profiles. Comparing myrtle populations under greenhouse conditions to wild myrtle, a research highlight in this study was the high concentrations of α-pinene, linalyl acetate, caryophyllene oxide, germacrene D, α-humulene, 1,8-cineole, limonene, and *p*-cymene. These components are likely to make myrtle EO useful for various applications. According to the results of the HCA, the myrtle populations in this research were mostly divided into four groups, including I: caryophyllene oxide/germacrene D/α-humulene/methyl eugenol chemotype; II: α-pinene/*p*-cymene/α-humulene and (*E*)-β-caryophyllene; III: α-pinene/1,8-cineole, and linalool; IV: linalyl acetate/γ-terpinene/1,8-cineole/limonene according to their primary EO components. The essential oil content of myrtle populations was influenced by a number of variables, such as geographic origin, environment, and genetic factors. The selections of myrtle populations, based on their significant chemical profiles, can assist breeders in developing unique plans for generating new genotypes and foregrounding their commercial cultivation.

## Figures and Tables

**Table 1:** Essential oils content and composition of 14 superior myrtle accessions under greenhouse conditions.

Compound	RI ^a^	RI ^b^	KH1	KH3	AT1	AT2	AT3	AT4	AT5	BN1	BN2	BN5	KA1	KA2	KA3	KA4
α-Pinene	936	939	3.21	16.28	37.44	53.09	2.35	2.63	25.00	5.10	11.26	17.28	31.23	6.95	25.93	30.64
*o*-Cymene	1022	1022	-	8.05	-	-	-	-	0.65	3.96	1.05	-	0.73	-	0.92	1.26
*p*-Cymene	1026	1024	-	13.18	-	0.72	-	-	0.78	4.76	-	-	0.78	-	-	-
Limonene	1029	1029	-	3.44	2.51	2.27	-	-	1.64	-	17.38	15.64	2.29	12.03	2.53	2.92
1,8-Cineole	1031	1031	18.04	3.96	7.60	6.74	2.02	-	4.91	-	14.43	7.16	13.19	7.52	17.43	17.64
(*E*)-β-Ocimene	1044	1050	-	-	-	-	-	-	0.46	-	-	-	-	-	-	-
γ-Terpinene	1056	1059	-	8.35	-	-	-	-	0.45	4.40	0.81	-	0.48	-	-	-
Linalool	1098	1096	24.70	-	16.83	13.78	24.97	-	19.57	13.10	23.15	24.89	17.70	23.69	18.74	20.53
α-Terpineol	1190	1188	7.37	5.02	4.092	0.57	3.68	1.14	2.44	3.99	4.016	2.70	3.17	4.11	3.69	3.29
Geraniol	1253	1252	5.028	3.63	1.84	0.79	-	6.56	1.73	2.99	1.11	-	1.74	2.76	2.16	2.018
Linalyl acetate	1258	1257	19.08	1.66	10.09	-	45.29	-	23.10	18.28	13.45	16.97	16.83	22.21	14.56	12.05
α-Terpinyl acetate	1347	1349	5.78	-	1.16	0.54	-	3.81	0.97	4.51	1.90	0.78	1.69	2.19	2.11	1.81
Neryl acetate	1361	1361	-	-	-	11.48	-	-	0.81	-	0.92	0.99	0.77	1.42	-	-
Geranyl acetate	1381	1381	6.99	5.90	2.75	2.14	6.41	4.38	2.99	10.90	4.40	3.89	2.02	3.91	2.25	1.74
Methyl eugenol	1402	1403	-	-	1.55	-	-	12.23	1.04	4.99	-	0.92	-	-	-	-
(*E*)-β-Damascone	1413	1413	5.32	2.96	3.67	1.09	3.34	4.46	1.77	5.35		1.18		2.74	1.87	-
(*E*)-β-Caryophyllene	1417	1417	-	8.90	2.41	2.17	5.86	-	2.97	4.96	1.10	0.71	1.42	1.20	1.66	-
α-Humulene	1450	1452	-	9.84	1.80	0.50	3.46	18.97	2.80	3.98	0.76	0.45	1.10	-	0.89	0.82
Germacrene D	1481	1482	-	-	2.95	-	-	19.19	-	-	-	-	-	2.74	-	-
α-Amorphene	1485	1483	-	-	-	1.15	-	-	-	-	-	3.23	-	-	2.67	1.01
(*E*)-Methyl isoeugenol	1495	1492	-	3.63	-	-	-	4.42	3.98	-	-	-	-	-	-	-
Caryophyllene oxide	1581	1583	4.02	5.02	2.42	0.56	2.58	21.80	1.04	8.10	4.25	0.97	1.51	4.61	1.48	2.25
Total			99.54	99.83	99.12	97.59	99.96	99.59	99.104	99.37	99.98	97.76	96.65	98.09	98.89	97.98
EO (%)			0.56	0.45	0.60	0.52	0.57	0.61	0.55	0.44	0.42	2.62	0.95	1.50	0.87	0.50

RI ^a^: Retention indices analyses on HP-5MS column.: Not detected compounds. RI ^b^: Retention index value taken from ADAMS library EO: Essential oil content ((g oil per 100 g of plant material) *w*/*w*%).

**Table 2:** Collection site and geographical characteristics of 14 superior myrtle accessions.

No.	Accession Names	Collection Site	Latitude	Longitude	Altitude (m)
1–2	KH1-KH3	Khergheh, Firozabad, Fars, Iran	285,345.7 N	522,240.3 E	1497
3–6	KA1-KA2-KA3-KA4	Kavar, Fars, Iran	290,600.4 N	524,811.1 E	1525
7–11	AT1-AT2-AT3-AT4-AT5	Atashkadeh, Fars, Iran	290,808.1 N	533,712.8 E	1478
12–14	BN1-BN2-BN5	Bagh nari, Noorabad mamasani, Fars, Iran	301,056.4 N	514,623.5 E	1293
